# Interactions among Vascular-Tone Modulators Contribute to High Altitude Pulmonary Edema and Augmented Vasoreactivity in Highlanders

**DOI:** 10.1371/journal.pone.0044049

**Published:** 2012-09-11

**Authors:** Zahara Ali, Aastha Mishra, Rahul Kumar, Perwez Alam, Priyanka Pandey, Rekhbala Ram, Tashi Thinlas, Ghulam Mohammad, M. A. Qadar Pasha

**Affiliations:** 1 Institute of Genomics and Integrative Biology, Delhi, India; 2 Department of Biotechnology, University of Pune, Pune, India; 3 Department of Medicine, SNM Hospital, Leh, Ladakh, Jammu and Kashmi, India; UT MD Anderson Cancer Center, United States of America

## Abstract

**Background:**

The interactions among various biomarkers remained unexplored under the stressful environment of high-altitude. Present study evaluated interactions among biomarkers to study susceptibility for high altitude pulmonary edema (HAPE) in HAPE-patients (HAPE-p) and adaptation in highland natives (HLs); both in comparison to HAPE-free sojourners (HAPE-f).

**Methodology/Principal Findings:**

All the subjects were recruited at 3500 m. We measured clinical parameters, biochemical levels in plasma and gene expression using RNA from blood; analyzed various correlations between and among the clinical parameters, especially arterial oxygen saturation (SaO_2_) and mean arterial pressure (MAP) and biochemical parameters like, asymmetric dimethylarginine (ADMA), serotonin (5-HT), 8-iso-prostaglandin F2α (8-isoPGF2α), endothelin-1 (ET-1), plasma renin activity (PRA), plasma aldosterone concentration (PAC), superoxide dismutase (SOD) and nitric oxide (NO) in HAPE-p, HAPE-f and HLs. ADMA, 5-HT, 8-isoPGF2α, ET-1 levels, and PAC were significantly higher (p<0.0001, each), whereas SOD activity and NO level were significantly lower in HAPE-p than HAPE-f (p≤0.001). Furthermore, ADMA, 5-HT, 8-isoPGF2α, NO levels and PAC were significantly higher (p<0.0001), whereas ET-1 level significantly (p<0.0001) and SOD activity non-significantly (p>0.05) lower in HLs than HAPE-f. The expression of respective genes differed in the three groups. In the correlations, SaO_2_ inversely correlated with ADMA, 5-HT and 8-isoPGF2α and positively with SOD in HAPE-p (p≤0.009). MAP correlated positively with 5-HT and 8-isoPGF2α in HAPE-p and HLs (p≤0.004). A strong positive correlation was observed between ADMA and 5-HT, 5-HT and 8-isoPGF2α (p≤0.001), whereas inverse correlation of SOD with ET-1 in HAPE-p and HLs (p≤0.004), with 5-HT and 8-isoPGF2α in HAPE-p (p = 0.01) and with 5-HT in HLs (p = 0.05).

**Conclusions/Significance:**

The interactions among these markers confer enhanced vascular activity in HLs and HAPE in sojourners.

## Introduction

The relevance of hypobaric hypoxia environment at high-altitude does not confine to adaptation but it also provides an excellent set up for studying pathophysiological conditions that result into various disorders [1]–[3]. Among these disorders high-altitude pulmonary edema (HAPE), a rare but fatal disorder, develops in susceptible sojourners upon rapid ascent to or physical exertion at altitudes above 2500 m [4]. HAPE, a non-cardiogenic pulmonary edema, is characterized by exaggerated pulmonary hypertension, which leads to vascular leakage through overperfusion and stress failure or both [4].

Different physiological pathways are activated under this environment that incorporate cellular changes like pulmonary vasoconstriction response, erythropoiesis and increased blood flow [1]–[2], [5], however several of the constituents of these pathways have not been evaluated. For example asymmetric dimethylarginine (ADMA), an endogenous nitric oxide synthase inhibitor, is an endothelial dysfunction and oxidative-stress marker [6]–[8]. It is crucially involved in various cardiovascular diseases [9] but its role in HAPE is yet to be established. Likewise, serotonin also known as 5-hydroxytryptamine, (5-HT), a mitogenic marker [10], [11] and F-iso-prostaglandins, the lipid peroxidation markers [12], [13] have not been explored in HAPE pathophysiology. Beside, the mitogenic and vasoconstrictory biomolecule, endothelin-1 (ET-1) is believed to have a role in HAPE pathogenesis [14]–[16]. The other constituents of vascular homeostasis cascade such as, plasma renin activity (PRA), angiotensin-1converting enzyme (ACE) and plasma aldosterone concentration (PAC) find their relevance because of their involvement in blood pressure regulation and fluid retention [17]–[19]. Equally imperative is the function of nitric oxide (NO) and antioxidants such as superoxide dismutase (SOD) that maintain vasodilatation and oxygenation at HA [20]–[23].

The constituents of vascular homeostasis, thus, become pertinent at HA. Although the basic effects of most of the molecules are known, the normal physiological or pathophysiological changes cannot be credited to an individual molecule; hence, we hypothesized that cross-talk among several molecules of different pathways may lead to HA disorders and adaptation.

To test our hypothesis, we set forth objectives to measure the biochemical parameters like ADMA, 5-HT, 8-isoPGF2α, and also ET-1, PRA, PAC, SOD and NO; to quantify transcriptional activity; importantly, to evaluate the various correlations between and among these molecules and clinical parameters; and, to evaluate association of each parameter with susceptibility to HAPE and their plausible role in adaptation. The study was performed in three well-defined groups, viz. HAPE-patients (HAPE-p), HAPE-free sojourners (HAPE-f) and highland natives (HLs; residing at heights ≥3500 m).

## Materials and Methods

### Study subjects

Prior to participation in the study, written informed consent was obtained from each participant. The subjects were categorized into three well-defined groups: 1) HAPE-patients (HAPE-p), n = 200, are sojourners who suffered the disorder upon exposure to HA (∼3500 m); 2) HAPE-free sojourners (HAPE-f), n = 200, are the healthy subjects, who visited HA under similar conditions but did not suffer from the disorder, and 3) healthy highland natives (HLs), n = 450, are the permanent residents to HA, residing at and above an altitude of 3500 m. The HLs are of Tibeto-Burman ethnicity residing at HA for many generations, and are declared fit and healthy. All the subjects were recruited through SNM hospital, Leh (∼3500 m), India. The two human ethical committees namely; ‘the human ethical committee of Institute of Genomics and Integrative Biology, Delhi’ and ‘the human ethical committee of Sonam Norboo Memorial Hospital, Leh, Ladakh’ had approved the investigation. The experimental part was performed in our laboratory in Delhi.

### Selection criteria

Diagnosis of HAPE was based on chest radiography that revealed infiltrates consistent with pulmonary edema. Pulmonary artery systolic-pressure (PASP) was measured in HAPE-p and HAPE-f using Sonos-5500 echocardiography (Hewlett-Packard, USA). The other clinical symptoms included hypoxemia, cough and dyspnea at rest, presence of pulmonary rales and cyanosis. Of note, HAPE-p acquired the disease upon first exposure to hypobaric hypoxia at 3500 m, whereas HAPE-f visited HA more than once (59%) and none had suffered from HA related disorders. The clinical parameters e.g., body mass index (BMI), blood pressure (BP), SaO_2_ and pulse rate (PR) were also measured. The subjects were given rest prior to BP measurement. Three measurements of BP, in supine position, using a calibrated mercury sphygmomanometer with appropriate adult cuff size were recorded. The SaO_2_ and PR were measured using Finger-Pulse Oximeter 503 (Criticare Systems Inc, USA). Any previous history of cardiopulmonary and other diseases was ruled out through questionnaire in the three groups. Lake Louise scoring was applied to rule out any symptoms of acute mountain sickness among HAPE-f.

### Biomarker estimation

Blood samples were collected at 3500 m. Ten ml of venous blood was drawn in acid-citrate-dextrose anticoagulant, plasma was collected and stored at −80°C until analyzed. Blood in case of patients was collected once HAPE was diagnosed. The blood from the subjects of the two healthy groups was drawn following overnight fasting.

ELISA was used to measure plasma ADMA (Uscn Life Science Inc, USA), 5-HT, 8-isoPGF2α and ET-1 levels (Assay Designs, USA). PRA and PAC were measured by radioimmunoassay (Immunotech, France) on a gamma counter (Ria Calc Wizard 1470, USA). SOD was estimated by the method of Kono et al. (1978), whereas NO was estimated by an enzymatic assay kit (Cayman Chemical, USA). Barring the radioimmunoassay, all the other measurements were performed on a high-throughput SpectraMax plus384 spectrophotometer (Molecular Devices, USA). The inter- and intra-assay coefficient of variations were less than 5% for ADMA, 5-HT, SOD and NO, whereas they were less than 10% for 8-isoPGF2α, ET-1, PRA and PAC.

### Gene expression

Total RNA was extracted from 2 ml whole blood by TRI reagent RT blood (Molecular Research Centre, Inc., USA). RNA quality and quantity was determined using a NanoDrop Technologies (ND-1000) spectrophotometer (Thermo Fisher Scientific, USA) and integrity was checked by running on 1.5% agarose gel. Total RNA, 1.5 µg, was used to generate cDNA by EZ-first strand cDNA synthesis kit (Biological Industries, Israel). The gene expression analysis for tryptophan hydroxylase-1 (*TPH-1*), gene for 5-HT, endothelin-1 (*ET-1*), renin (*REN*), cytochrome P450, family 11, subfamily B, polypeptide 2 (*CYP11B2*), superoxide dismutase (*SOD*) and nitric oxide synthase 3 (*NOS3*) was performed on ten samples each of HAPE-p, HAPE-f and HLs. Primers for real-time PCR were designed using the Pearl Primer software (ver. 1.1.20) and are listed in Table S1. Real-time PCR was performed in duplicate and repeated thrice for each gene and each sample on an ABI Prism 7300 Sequence Detection System (Applied Biosystems, USA) using SYBR Green PCR Master Mix (Applied Biosystems, USA). Relative transcript quantities were calculated using the ΔΔCt method with 18S rRNA as the endogenous reference.

### Correlation analyses

Four types of correlations were analyzed between, 1) clinical parameters; SaO_2_ and MAP, 2) biomarkers; seven biomarkers namely, ADMA, 5-HT, 8-isoPGF2α, ET-1, PRA, PAC and SOD were correlated with each other using regression model, 3) next, the clinical parameters were tested against the seven biomarkers and finally, 4) the comparison was made between these biomarkers with respective gene expression.

### Statistical analyses

All the values are presented as mean ± SD. Data were analyzed using a statistical package for social sciences for windows (SPSS ver. 15.0) and EPIINFO ver. 6.0 software. Unpaired Student's *t*-test with two tailed values was performed to compare the differences between the two groups. Log transformed 5-HT and 8-isoPGF2α levels were used for the analysis. Difference in 5-HT and 8-isoPGF2α levels between the three groups was analyzed by one-way analysis of variance (ANOVA). The regression model was used to witness the overall correlation among biomarkers and clinical parameters. The p value was calculated by analysis of covariance method (ANCOVA) after adjustment with the covariates age, gender and BMI followed by Bonferroni's correction test. A p value of <0.05 was considered statistically significant.

## Results

### Clinical characteristics

Table 1 portrays the clinical characteristics of HAPE-p versus HAPE-f and HLs versus HAPE-f. The systolic blood pressure (SBP), mean arterial pressure (MAP), respiratory rate (RR), pulse rate (PR), SaO_2_ and PASP differed between HAPE-p and HAPE-f (p<0.05), and SBP, DBP, MAP and SaO_2_ between HLs and HAPE-f (p<0.05). Of note, SaO_2_ at 69±13.5% was the lowest in HAPE-p (p<0.0001); the decrease in SaO_2_ in patients was almost 20% of the average SaO_2_ of HAPE-f. Interestingly, SaO_2_ at 87±5.6% was also lower in HLs as against the 91±3.9% in HAPE-f (p<0.0001). PASP was 1.73 times higher in HAPE-p than HAPE-f (p<0.0001). Table 2 represents the baseline characteristics of HAPE-p versus HAPE-f and HLs versus HAPE-f. The height, weight, and BMI differed between the respective groups (p<0.05).

**Table 1:** Clinical characteristics of the HAPE-p, HAPE-f and HLs.

Clinical characteristics	HAPE-p	HAPE-f	HLs	p value	
				HAPE-p vs HAPE-f	HLs vs HAPE-f
n	200	200	450		
SBP, mmHg	124.6±21.1	119.8±3.5	128.1±24.0	0.007	<0.0001
DBP, mmHg	82.7±17.0	80.4±10.0	84.1±15.0	0.099	0.001
MAP, mmHg	96.7±16.0	93.5±10.0	96.4±10.0	0.016	0.0006
RR, rate/min	24.7±6.2	22.6±3.2	-	<0.0001	-
PR, rate/min	92.2±22.9	80.1±19.9	84.0±15.7	<0.0001	0.007
SaO_2_, %	69.0±13.5	91.0±3.9	87.0±5.6	<0.0001	<0.0001
PASP, mmHg	45.0±8.3	25.9±1.1	-	<0.0001	-

Data are presented as mean ± standard deviation and are compared by Student's *t*-test. n, number of subjects; SBP, systolic blood pressure; DBP, diastolic blood pressure; MAP, mean arterial pressure; RR, respiratory rate; PR, pulse rate; SaO_2_, arterial oxygen saturation; PASP, pulmonary artery systolic pressure.

**Table 2:** Baseline characteristics of the HAPE-p, HAPE-f and HLs.

				p value	
Baseline characteristics	HAPE-p	HAPE-f	HLs	HAPE-p vs HAPE-f	HLs vs HAPE-f
n	200	200	450		
Age, yrs	27.7±8.5	26.16±5.8	36.0±2.1	0.03	<0.0001
Height, cm	166.0±7.1	162.0±6.9	159.0±5.1	<0.0001	<0.0001
Weight, Kg	60.0±10.0	50.0±7.1	52.0±6.1	<0.0001	0.0002
BMI, Kg/m^2^	21.1±3.8	19.22±2.7	20.3±2.6	<0.0001	<0.0001
Number of visits	≤1	≥3	Residents		

Data are presented as mean ± standard deviation and are compared by Student's *t*-test. n, number of subjects; BMI, body mass index.

### Biomarker status

Table 3 represents the status of biomarkers in HAPE-p against HAPE-f and HLs against HAPE-f. Significantly higher levels of ADMA, 5-HT, 8-isoPGF2α, ET-1, PAC (p<0.0001, each) and PRA (p = 0.02); whereas, significantly lower SOD activity and NO level were obtained in HAPE-p than HAPE-f (p≤0.001). In terms of fold difference, the levels of ADMA, 5-HT, 8-isoPGF2α, ET-1, PRA and PAC were higher by 4.19, 1.5, 1.5, 2.3, 2.3 and 2.0 times, while SOD activity and NO level were lower by 1.7 and 1.27 times, respectively, in HAPE-p.

**Table 3:** Plasma levels of biomarkers in HAPE-p and HAPE–f and HLs.

Biomarkers	HAPE-p	HAPE-f	HLs	p value	
				HAPE-p vs HAPE-f	HLs vs HAPE-f
ADMA, nmol/mL	4.57±2.92 (200)	1.09±0.46 (200)	1.88±0.82 (200)	<0.0001	<0.0001
5-HT, ng/mL	5.50±1.10 (200)	3.60±1.40 (200)	4.30±2.20 (450)	<0.0001	<0.0001
8-iso PGF2α, pg/mL	8.90±1.60 (200)	6.04±0.89 (200)	7.90±0.73 (450)	<0.0001	<0.0001
ET-1, pg/mL	8.10±2.50 (63)	3.60±0.870 (200)	1.72±0.95 (200)	<0.0001	<0.0001
PRA, ng/ml/h	1.64±0.95 (63)	0.70±0.12 (200)	0.95±0.21 (200)	0.02	0.04
PAC, pmol/L	318.6±275 (63)	166.9±98.0 (200)	268.6±120 (200)	<0.0001	<0.0001
SOD, U/mL	0.84±0.41 (200)	1.40±0.360 (200)	1.22±0.52 (450)	0.001	0.40
NO, µM/L	57.67±30.12 (200)	73.75±35.26 (200)	122.4±70.0 (200)	<0.0001	<0.0001

Data are presented as mean ± standard deviation and are compared by one–way ANOVA. n, number of subjects; ADMA, asymmetric dimethylarginine; 5-HT, serotonin; 8-isoPGF2α, 8-iso-prostaglandin-F2α; ET-1, endothelin-1; PRA, plasma renin activity; PAC, plasma aldosterone concentration; SOD, superoxide dismutase; NO, nitric oxide. The p values are adjusted with age, gender and BMI.

As can be seen from table 3, significantly higher levels of ADMA, 5-HT, 8-isoPGF2α, PAC, NO (p<0.0001, each) and PRA (p = 0.04) were obtained in HLs than HAPE-f; contrary to this, significantly lower ET-1 level and lower SOD activity were obtained in HLs (p<0.0001 and p = 0.40, respectively). In terms of fold difference, the levels of ADMA, 5-HT, 8-isoPGF2α, PRA, PAC and NO were higher by 1.72, 1.2, 1.3, 1.4, 2.0 and 1.65 times, respectively, when compared with HAPE-f, whereas ET-1 level and SOD activity were lower by 2.1 and 1.2 times, respectively, in HLs than HAPE-f.

### Gene expression

**Figure 1:**
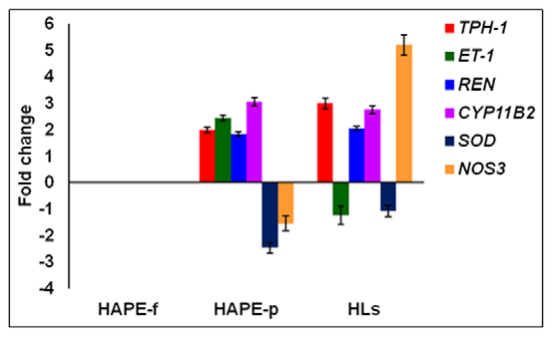
Relative gene expression by real-time PCR expressed as fold change in HAPE-p and HLs. Bars (mean ± SD) above the base line show upregulation and bars below the base line show downregulation. The differential expression (+/−) varied between 1.01–5.2. *TPH-1*, tryptophan hydroxylase-1; *ET-1*, endothelin-1; *REN*, renin; *CYP11B2*, cytochrome P450, family 11, subfamily B, polypeptide 2; *SOD*, superoxide dismutase; *NOS3*, nitric oxide synthase 3; HAPE-p, high altitude pulmonary edema patients; HLs, healthy highland natives; HAPE-f, High altitude pulmonary edema free sojourners. The comparisons of HAPE-p and HLs were made against HAPE-f.

Figure 1 represents the expression pattern of *TPH-1*, *ET-1*, *REN*, *CYP11B2*, *SOD* and *NOS3*. In HAPE-p, the expression of *TPH-1*, *ET-1*, *CYP11B2* and *REN* was up-regulated by 2.0, 2.45, 3.06 and 1.84 fold (p = 0.01, p = 0.015, p = 0.032 and p>0.05, respectively), whereas the expression of *SOD* and *NOS3* was down-regulated by 2.46 and 1.55 fold, respectively, against HAPE-f (p = 0.01, p>0.05). In HLs, the expression of *TPH-1*, *CYP11B2*, *NOS3* and *REN* was up-regulated by 3.0, 2.76, 5.2 and 2.05 fold (p = 0.001, p = 0.023, p = 0.001 and p>0.05), against HAPE-f, respectively, whereas *ET-1* and *SOD* was down-regulated by 1.24 and 1.07 fold (p>0.05), respectively, against HAPE-f.

### Correlations, the extent of interactions

**Figure 2:**
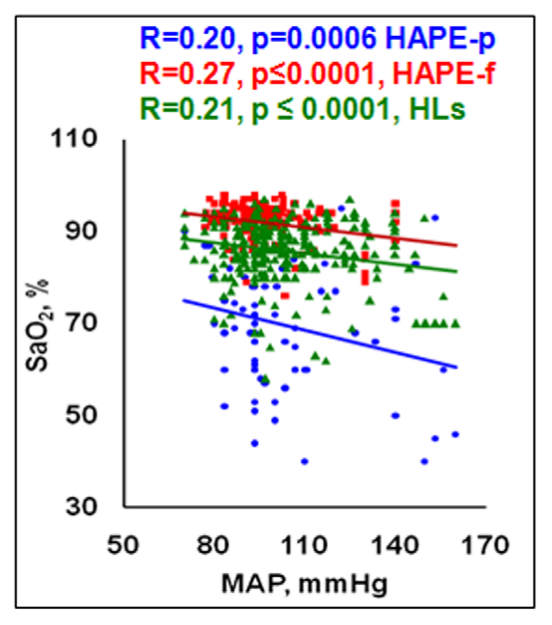
Clinical correlations in the three groups i.e. HAPE-p, HAPE-f and HLs. An inverse correlation was obtained between the clinical parameters viz MAP and SaO_2_ in the three groups; MAP, mmHg; SaO_2_, %.

Figure 2 and Figure S1, S2, S3, S4 represent various correlation analyses in the three studied groups.

### Correlations between clinical parameters

A strong inverse correlation was observed between SaO_2_ and MAP in HAPE-p (p = 0.0006), HAPE-f (p≤0.0001) and HLs (p≤0.0001) as can be seen from figure 2.

### Correlations between Biomarkers

Here, Figure S1, represents nine correlation comparisons between the biomarkers except NO, which has been presented in Figure S2. A strong positive correlation was observed between ADMA and 5-HT in HAPE-p and HAPE-f (Figure S1 a), ADMA also showed an inverse correlation with SOD in HAPE-p and HAPE-f (p≤0.0001; Figure S1b). ADMA also showed a marginal correlation with 8-isoPGF2α only in HLs (p = 0.04; Figure S1c). 5-HT showed a strong positive correlation with 8-isoPGF2α in the three groups (p≤0.001; Figure S1d) and with ET-1 in HLs (p = 0.03, Figure S1e); while, it inversely correlated with SOD in HLs (p = 0.05; Figure S1f). Although, 8-isoPGF2α was positively correlated with 5-HT (p≤0.001; Figure S1d), it did not correlate with ET-1 in the three groups (p>0.05; Figure S1g), whereas, it inversely correlated with SOD in HAPE-p and HAPE-f (p = 0.01; Figure S1h). ET-1 showed significant inverse correlation with SOD in HAPE-p and HLs (p≤0.004; Figure S1i). In addition, NO was inversely correlated with ADMA, 5-HT and 8-isoPGF2α in the three groups (p≤0.031, p≤0.06, and p≤0.022, respectively, Figure S2). The correlation outcome on renin and aldosterone was not encouraging hence it is not included here.

### Correlations between clinical parameters and Biomarkers

Here, seven correlation comparisons are provided, between clinical parameters and biomarkers in Figure S3, and between clinical parameters and NO in Figure S4. SaO_2_ showed inverse correlation with ADMA in the three groups (p≤0.0084; Figure S3a), with 5-HT and 8-isoPGF2α in HAPE-p (p = 0.0001; Figure S3b; p = 0.0003; Figure S3c, respectively); whereas, it was positively correlated with SOD in HAPE-p (p = 0.009; Figure S3d), and with NO in the three groups (p≤0.022; Figure S4a). MAP showed significant positive correlation with ADMA in HAPE-p (p<0.05; Figure S3e), with 5-HT in the three studied groups (p = 0.0001; Figure S3f), with 8-isoPGF2α in HAPE-p and HLs (p≤0.004; Figure S3g) and inversely with NO in the three groups (p≤0.02; Figure S4b).

### Correlation between Biomarkers and Gene expression

The correlation comparison between biomarkers and the respective gene expression profile was evaluated. Plasma 5-HT concentration and *TPH-1* gene expression were upregulated by 1.5 and 2.0 fold, respectively, in HAPE-p, and 1.2 and 3.0 fold, respectively, in HLs. Plasma ET-1 concentration and *ET-1* gene expression in HAPE-p were upregulated by 2.3 and 2.45 fold, respectively, whereas in HLs both were downregulated by 2.1 and 1.24 fold, respectively. Plasma renin and aldosterone concentration and gene expression in HAPE-p were upregulated by 2.3, 1.84 and 2.0, 3.06 fold, respectively; also in HLs, the two were upregulated by 1.4, 2.05 and 2.0, 2.76 fold, respectively. Contrary to these findings, plasma SOD activity and *SOD* gene expression were downregulated by 1.7 and 2.46 fold, respectively, in HAPE-p; it followed with 1.2 and 1.07 fold downregulation, respectively, in HLs. A decrease of 1.27 and 1.55 fold in plasma NO and NOS3 gene expression was observed in HAPE-p, whereas the fold changes were elevated by 1.65 and 5.2 fold in plasma and gene expression, respectively, in HLs. This comparison compliments the biolevels and its respective gene expression.

### The multiple correlations

**Figure 3:**
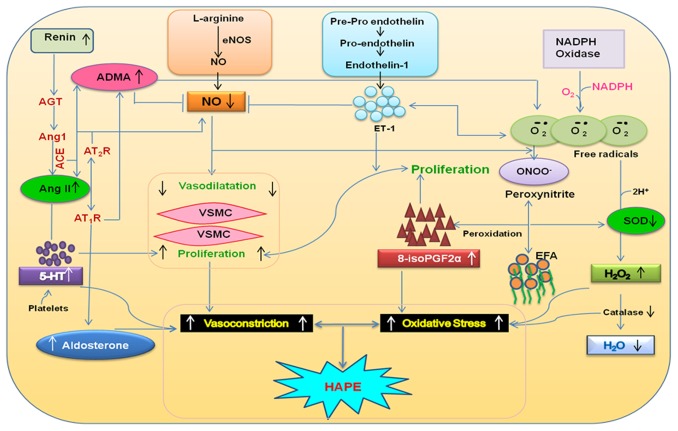
Schematic presentation of the studied biomarkers in a physiological function under hypobaric hypoxia. Interactions of several of these biomarkers that translate into the incidence of HAPE are also interpretable through the renin–angiotensin–aldosterone system, Kinin–kallikrein system, and the pathways of ET-1, 5-HT, NO signaling and oxidative-stress. ADMA, asymmetric dimethylarginine; ATII, angiotensin II; AT-1R, angiotensin-II type I receptor; AT-2R, angiotensin-II type II receptor; AngI, angiotensin I; AngII, angiotensin II; ACE, angiotensin-I converting enzyme; AGT, angiotensinogen; 8-isoPGF2α, 8-iso-prostaglandinF2α; ROS, reactive oxygen species; SOD, superoxide dismutase; O2˙-, superoxide anion; ONOO^−^, peroxynitrite; 5-HT, serotonin; ET-1, endothelin-1; ET-_A_ and ET-_B_, endothelin receptors A and B; NO, nitric oxide; NOS3, endothelial nitric oxide synthase; VSMC, vascular smooth muscle cell; H_2_O_2_, hydrogen peroxide; H_2_O, water; EFA, essential fatty acids.

The multiple regression analysis provided the depiction of interaction among these biomarkers in the etiology of HAPE and adaptation (Figure S5). Positive correlations were observed among the vasoconstrictors like ADMA, 5-HT, 8-isoPGF2α and ET-1, (p between 0.03–0.000) and inverse correlations of these vasoconstrictors were observed with SOD and NO (p value ranges from 0.066–0.000) in the three groups. Among the clinical parameters, MAP showed positive correlation with ADMA, 5-HT, 8-isoPGF2α and ET-1 (p>0.05) and inverse correlation with SOD and NO (p value ranges from 0.37–0.018). SaO_2_ correlated inversely with ADMA, 5-HT, 8-isoPGF2α and ET-1 (p value ranges from 0.05–0.001) and positively with SOD and NO (p value ranges from 0.4–0.02). The schematic interactions of the studied molecules are profiled in Figure 3. The interactions between these molecules clearly suggested impairment of the endothelial function and vascular homeostasis (Figure 3) thereby leading to HAPE.

## Discussion

The highlight of our study is the investigation of several biomarkers with respect to HAPE and adaptation; few biomarkers were investigated until now.

It was encouraging to note that these molecules provided a glimpse of individual and interactive-mode function and the phenotypic outcome. ADMA, one of the endothelial dysfunction and oxidative-stress markers was significantly elevated in HAPE. It is synthesized during the methylation of arginine residues in a protein, and is released during proteolysis. ADMA plays three crucial roles; it competitively inhibits the cellular L-arginine uptake [24], [25], thereby inhibiting endogenous NOS3, enhances ACE activity [26] and elevates reactive oxygen species [8], [26]. We figured out that all the three functions were involved in HAPE, because of the fact that NO levels were found consistently lower, whereas ACE activity and aldosterone levels and the oxidative stress markers were higher in the present and previous studies [14], [27]. It is known that in renin-angiotensin aldosterone system, angiotensin II produced by ACE stimulates aldosterone synthase to secrete aldosterone that regulates water and salt retention [28]; on the other hand, in kallikren-kinin system, ACE proteolyses bradykinin deterring NO production, the net result of this cascade being increased vascular resistance and high BP [29], [30]. Likewise, our observation of lower activity of SOD in the present study and higher level of 8-isoPGF2α [31] were reminiscent of dysfunction of the oxidative-stress pathway and its causative role in HAPE. In fact, the expression level of two related genes was in accordance to these markers such as higher expression of pro-oxidant gene, cytochrome b-245, alpha polypeptide and lower expression of another antioxidant gene, glutathione S-transferase pi 1 [31]. The formation of abnormal phospholipids [32] such as in the present situation apparently exerted profound effect on membrane fluidity and integrity, a sign of oxidant injury. The positive correlation of ADMA with 5-HT and ET-1 pointed to exaggerated vasoconstriction.

Though, these effects were more pronounced in HAPE, similar trends were noticed in natives, pointing to the fact that they were also under continuous stress but were more or less adapted to such a stressful environmental condition. It, importantly, points to the fact that chronic exposure results into elevated vascular reactivity but not as severely as in acute exposure in susceptible sojourners.

Another important finding was the elevated levels of 5-HT that underline its causative role in the pathogenesis of HAPE. 5-HT exerts mitogenic and co-mitogenic effects on pulmonary artery smooth muscle cells [10], [33]. The constricting action of 5-HT on smooth muscle cells is mainly mediated through G-protein coupled receptors, 5-HT_1B_ and 5-HT_2A_, and internalization by its transporter, which eventually potentiates PH and associated diseases [10], [34]–[35]. 5-HT is also involved in ROS generation through the interaction with NADPH oxidase, and proliferation through the interaction with angiotensin II [36], [37]. Although investigations have delineated the potential role of ADMA and 5-HT in the pathophysiology of PH [9]–[11], [38], [39], knowledge with respect to HAPE was acquired only through present study. Among the other physiological systems, ET-1 is a known contributor to the pathogenesis of HAPE [14]–[16]. Berger and colleagues (2009) had reported its involvement in PASP through hypoxia-induced pulmonary vasoconstriction [40], which is concurrent to our findings of elevated ET-1 and PASP in HAPE.

Equally pertinent would be to describe here the role of NO, which being a critical mediator of vasodilation remains cardinal to these physiological inferences. However, under the stressful environment of HA, several of the mediators of vasoconstriction interfere with the production of NO. True to this statement, we could observe inverse interaction of NO with vasoconstrictors; the net result was upsetting the normal physiological function.

It would further be pertinent to highlight the correlations between SaO_2_ and stress markers; the former is an index of oxygen affinity in blood at HA. An inverse correlation of SaO_2_ with MAP, ADMA, 5-HT and 8-isoPGF2α was an indication that the homeostasis of body had shifted towards disease susceptibility, which was observed in the patients. Higher oxygen availability in blood and controlled production of stress markers may prove fruitful in defining physical activity and even high endurance. This study, thus, imparts imperative knowledge about the SaO_2_ phenotype in relation to health and adaptation.

Finally, our evaluation of biomarkers in native population has been equally motivating and interesting. Although, Tibetans are one of the best-adapted populations, however, until present study literature was scant on the role of these biomarkers. It is of consequence to add that HLs revealed higher ADMA, 5-HT, 8-isoPGF2α, PRA and PAC and among the clinical parameters, relatively higher DBP, SBP and MAP. Yet, by virtue of their long term residence, any kind of negative consequences may have been counterbalanced by factors that maintained vascular tone at HA, such as reduced plasma ET-1 and higher NO and SaO_2_ levels as was observed in the present study, and as was reported earlier [41]–[43]. It is likely that the HLs have evolved few assorted mechanisms in response to hypoxia. Thus, a normal or enhanced vagal tone and preserved vasomotion in HLs are probably evidence of adaptation.

It may be of relevance to add on genetic aspects in relation to biomarkers in HAPE and adaptation. Last two decades focused on the candidate markers of vascular homeostasis pathway and oxidative stress. The notable genetic variants from among the several genes studied were those of angiotensin-I converting enzyme (*ACE*); angiotensinogen; aldosterone synthase; nitric oxide synthase 3 (*NOS3*); endothelin-1 (*ET-1*); β-adrenergic receptor type 2; cytochrome b-245 alpha polypeptide (*CYBA*) and glutathione S-transferase pi 1 (*GSTP1*). Several of the variants were reported to associate with adaptation and disorder. Interestingly, the variants were associated with the biolevels. Such as the *ACE* D allele of I/D polymorphism was associated with higher ACE activity and lower SaO_2_ level in patients or the I allele with lower activity and higher SaO_2_ level in healthy subjects [14], [43]; *ET-1* longer repeats of (CT)n-(CA)n and *NOS3* 4a allele of 4b/4a with lower levels of ET-1 and NO, respectively [14], [42]. *CYBA* A and T alleles of −930A/G and H72Y (C/T), *GSTP1* A and C alleles of I105V (A/G) and A114V (C/T) were associated with lower levels of 8-iso PGF2α [31]. A reverse trend was observed with the other alleles on the respective same loci. In recent years the oxygen sensing pathway has been reported to associate with adaptation in highlanders; the genome-wide association studies have revealed the involvement of the genes EGL nine homolog 1 and endothelial PAS domain protein 1 [44]–[46] in adaptation.

The prominent findings of the present study are the emergence of ADMA, 5-HT and 8-isoPGF2α as novel markers having promising role in the regulation of physiological functions at HA; as a result increased oxidative and pulmonary stress could be predicted. Furthermore it was also evident from our results that these biomarkers bonded together to produce the phenotypic outcome. The relative involvement, however, has to be evaluated through continued efforts. The added advantage of this study was that it was carried out in a fairly larger sample size. Among the limitations, PASP could not be measured in highlanders, as these being healthy native individuals did not consent to additional tests unless significantly required. PASP was measured in HAPE-p and HAPE-f using echocardiography and not through right heart catheterization, as the latter is mandatory for severe heart and pulmonary ailments like IPAH, FPAH etc and is a risky procedure thus is not permitted in SNM hospital. The two baseline characteristics, age and BMI need mention here. The HLs were older in age than the two other groups; however this age difference apparently did not influence the levels, although a younger age group would have validated this. The BMI in this context was in support of the above notion. Despite the age difference, the BMI was within the normal range as per national guidelines. Moreover, the two controls HAPE-p and HLs although revealed statistical difference but the mean BMI value was 19 and 20 kg/m^2^. Also BMI is not a phenotype of importance in HAPE hence in the present study BMI and age were used as confounders. Hence it seems these two parameters did not influence the biolevels. Nevertheless these parameters would be scrutinized in further studies.

## Supporting Information

Figure S1 **(a–i): Correlation analyses among biochemical parameters in the three groups i.e. HAPE-p, HAPE-f and HLs.** Proportional or inverse correlations were obtained between a) ADMA, nmol/mL and 5-HT, ng/mL; b) ADMA, nmol/mL and SOD, U/mL; c) ADMA, nmol/mL and 8-isoPGF2α, pg/mL; d) SOD, U/mL and ET-1, pg/mL; e) 5-HT, ng/mL and ET-1, pg/mL; f) and 8-isoPGF2α, pg/mL and ET-1, pg/mL; g) 8-isoPGF2α, pg/mL and 5-HT, ng/mL; h) SOD, U/mL and 5-HT, ng/mL; i) SOD, U/mL and 8-isoPGF2α, pg/mL.
(TIF)

Figure S2 **(a–e): Correlation analyses among biochemical parameters with NO in the three groups i.e. HAPE-p, HAPE-f and HLs.** Proportional or inverse correlations were obtained between a) NO, µmol/mL and ADMA, nmol/mL; b) NO, µmol/mL and 5-HT, ng/mL; c) NO, µmol/mL and 8-isoPGF2α, pg/mL; d) NO, µmol/mL and SOD, U/mL; e) NO, µmol/mL and ET-1, pg/mL.
(TIF)

Figure S3 **(a–g): Correlation analyses between biochemical parameters and clinical parameters in the three groups i.e. HAPE-p, HAPE-f and HLs.** Proportional or inverse correlations were obtained between a) ADMA, nmol/mL and SaO_2_, %; b) 5-HT, ng/mL and SaO_2_,%; c) 8-isoPGF2α, pg/mL and SaO_2_, %; d) SOD, U/mL and SaO_2_, %; e) ADMA, nmol/mL and MAP, mmHg; f) 8-isoPGF2α, pg/mL and MAP, mmHg; g) 5-HT, ng/mL and MAP, mmHg.
(TIF)

Figure S4 **(a–b): Correlation analyses between NO and clinical parameters in the three groups i.e. HAPE-p, HAPE-f and HLs.** Proportional or inverse correlations were obtained between a) NO, µmol/mL and SaO_2_, %; b) NO, µmol/mL and MAP, mmHg.
(TIF)

Figure S5 **(a–c): Multiple regression analysis in the three groups i.e. HAPE-p, HAPE-f and HLs.** The boxes with shades of red depicted positive correlations and boxes with shades of green depicted inverse correlations. Each box contains the coefficient of correlation along with p value. The p value was calculated placing one biomarker against all the studied biomarkers.
(TIF)

Table S1 **Real-Time PCR conditions for** ***TPH-1*** **,** ***ET-1*** **,** ***REN*** **,** ***CYP11B2*** **,** ***SOD*** **and** ***NOS3*** **.**
(DOC)
