# Supplementary figures and images for: Interactions among Vascular-Tone Modulators Contribute to High Altitude Pulmonary Edema and Augmented Vasoreactivity in Highlanders
Source: PLoS One. 2012 Sep 11;7(9):e44049. doi: 10.1371/journal.pone.0044049 (PMC3439466; doi:10.1371/journal.pone.0044049)

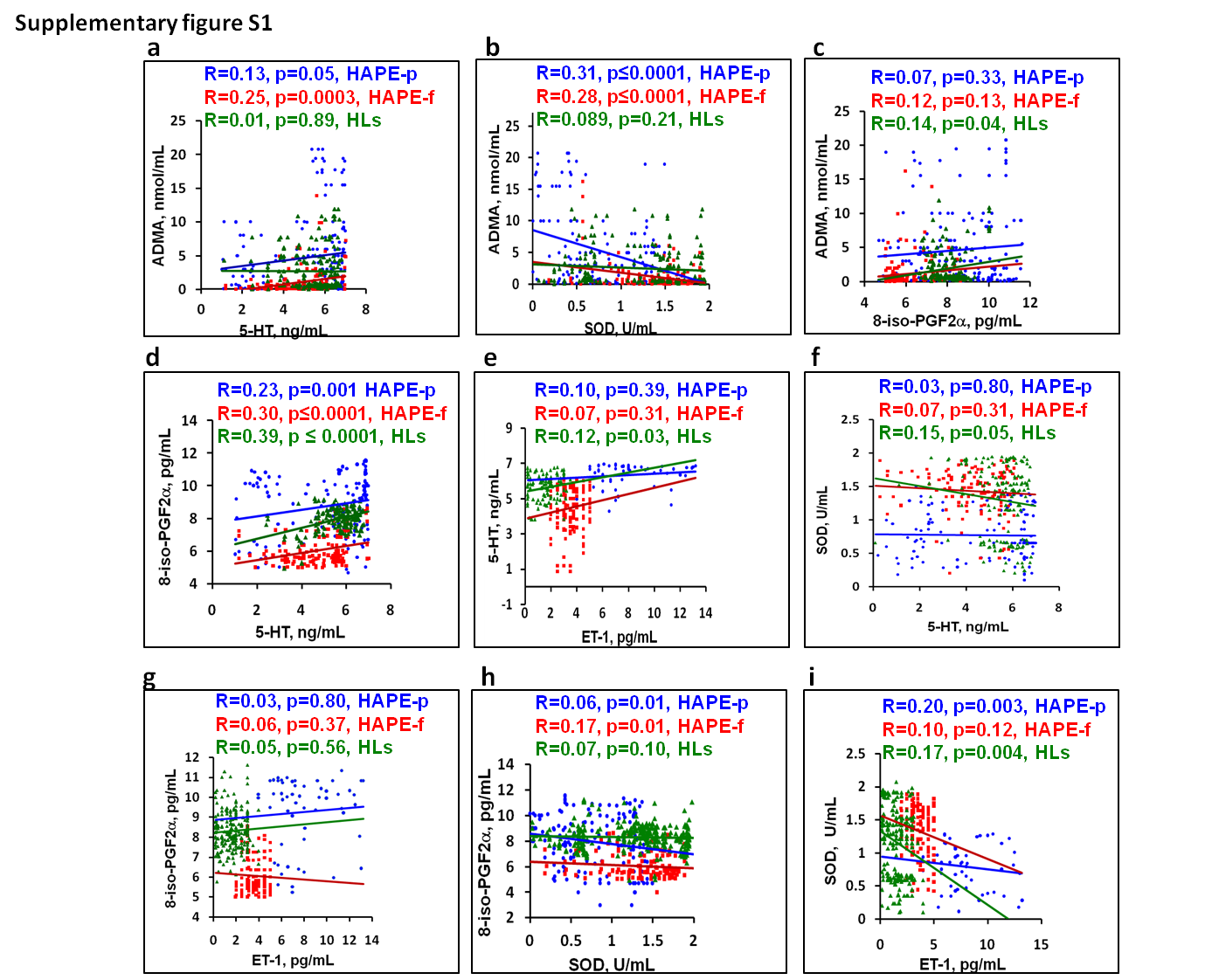

Supplement: Figure S1 — (a–i): Correlation analyses among biochemical parameters in the three groups i.e. HAPE-p, HAPE-f and HLs. Proportional or inverse correlations were obtained between a) ADMA, nmol/mL and 5-HT, ng/mL; b) ADMA, nmol/mL and SOD, U/mL; c) ADMA, nmol/mL and 8-isoPGF2α, pg/mL; d) SOD, U/mL and ET-1, pg/mL; e) 5-HT, ng/mL and ET-1, pg/mL; f) and 8-isoPGF2α, pg/mL and ET-1, pg/mL; g) 8-isoPGF2α, pg/mL and 5-HT, ng/mL; h) SOD, U/mL and 5-HT, ng/mL; i) SOD, U/mL and 8-isoPGF2α, pg/mL. (TIF) [file pone.0044049.s001.tif]

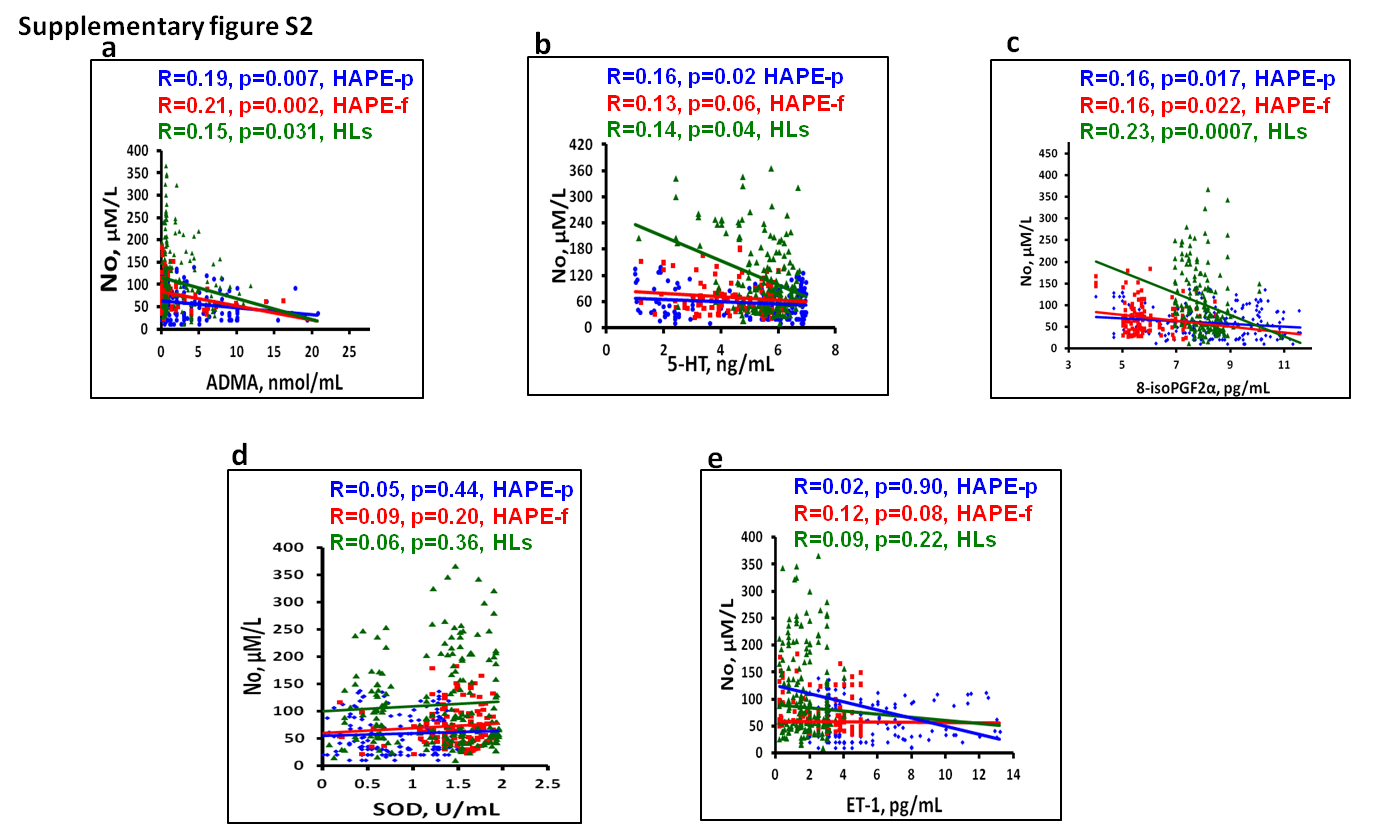

Supplement: Figure S2 — (a–e): Correlation analyses among biochemical parameters with NO in the three groups i.e. HAPE-p, HAPE-f and HLs. Proportional or inverse correlations were obtained between a) NO, µmol/mL and ADMA, nmol/mL; b) NO, µmol/mL and 5-HT, ng/mL; c) NO, µmol/mL and 8-isoPGF2α, pg/mL; d) NO, µmol/mL and SOD, U/mL; e) NO, µmol/mL and ET-1, pg/mL. (TIF) [file pone.0044049.s002.tif]

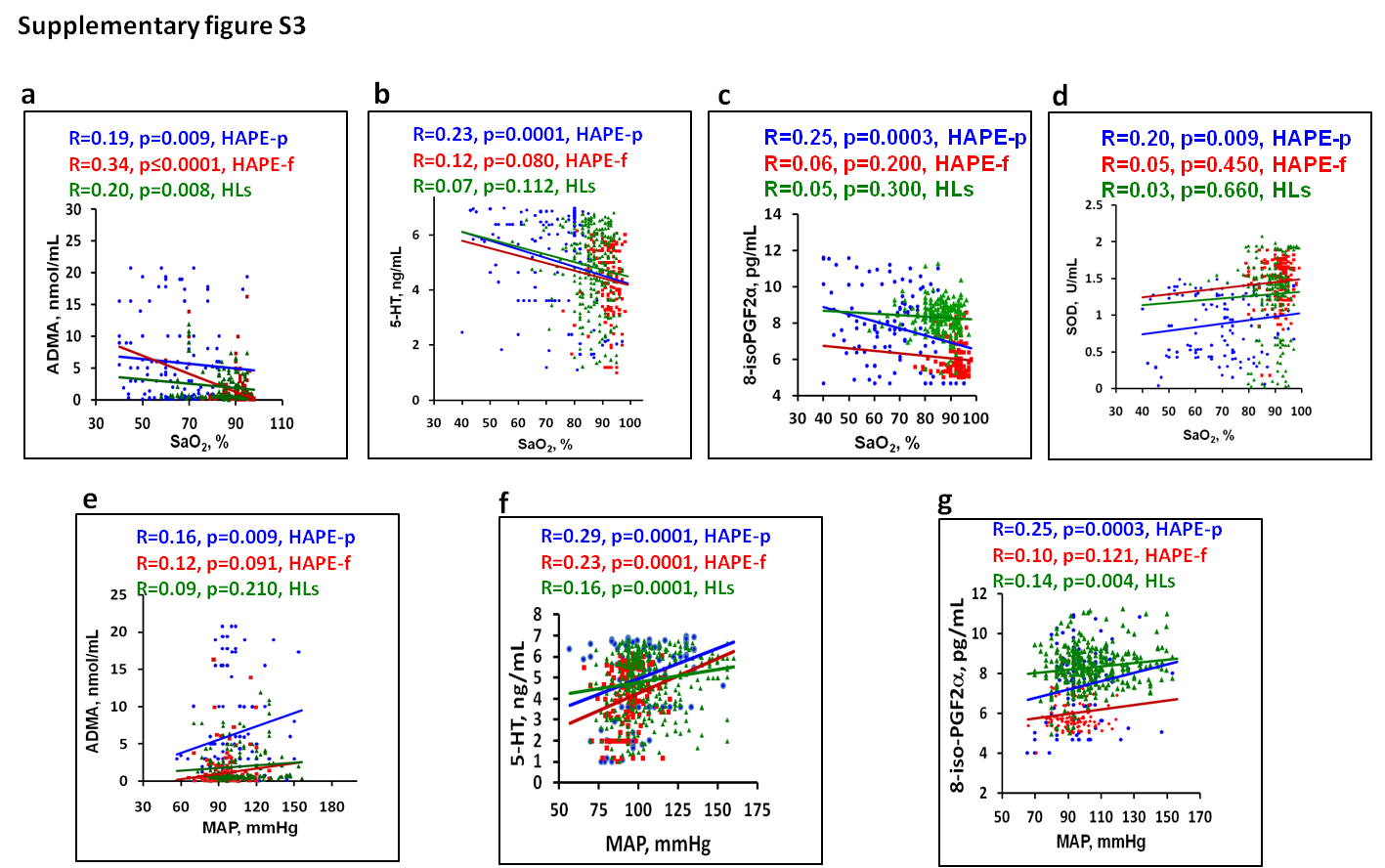

Supplement: Figure S3 — (a–g): Correlation analyses between biochemical parameters and clinical parameters in the three groups i.e. HAPE-p, HAPE-f and HLs. Proportional or inverse correlations were obtained between a) ADMA, nmol/mL and SaO2, %; b) 5-HT, ng/mL and SaO2,%; c) 8-isoPGF2α, pg/mL and SaO2, %; d) SOD, U/mL and SaO2, %; e) ADMA, nmol/mL and MAP, mmHg; f) 8-isoPGF2α, pg/mL and MAP, mmHg; g) 5-HT, ng/mL and MAP, mmHg. (TIF) [file pone.0044049.s003.tif]

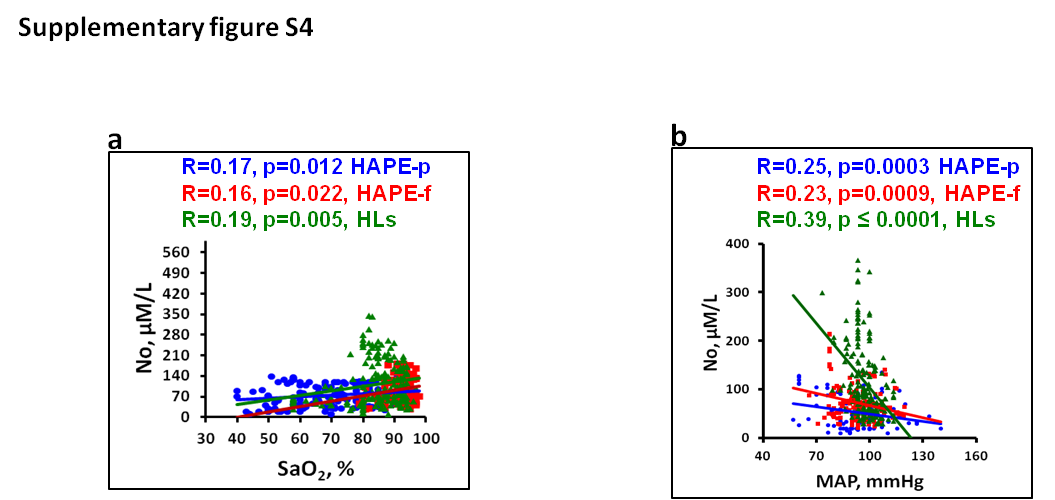

Supplement: Figure S4 — (a–b): Correlation analyses between NO and clinical parameters in the three groups i.e. HAPE-p, HAPE-f and HLs. Proportional or inverse correlations were obtained between a) NO, µmol/mL and SaO2, %; b) NO, µmol/mL and MAP, mmHg. (TIF) [file pone.0044049.s004.tif]

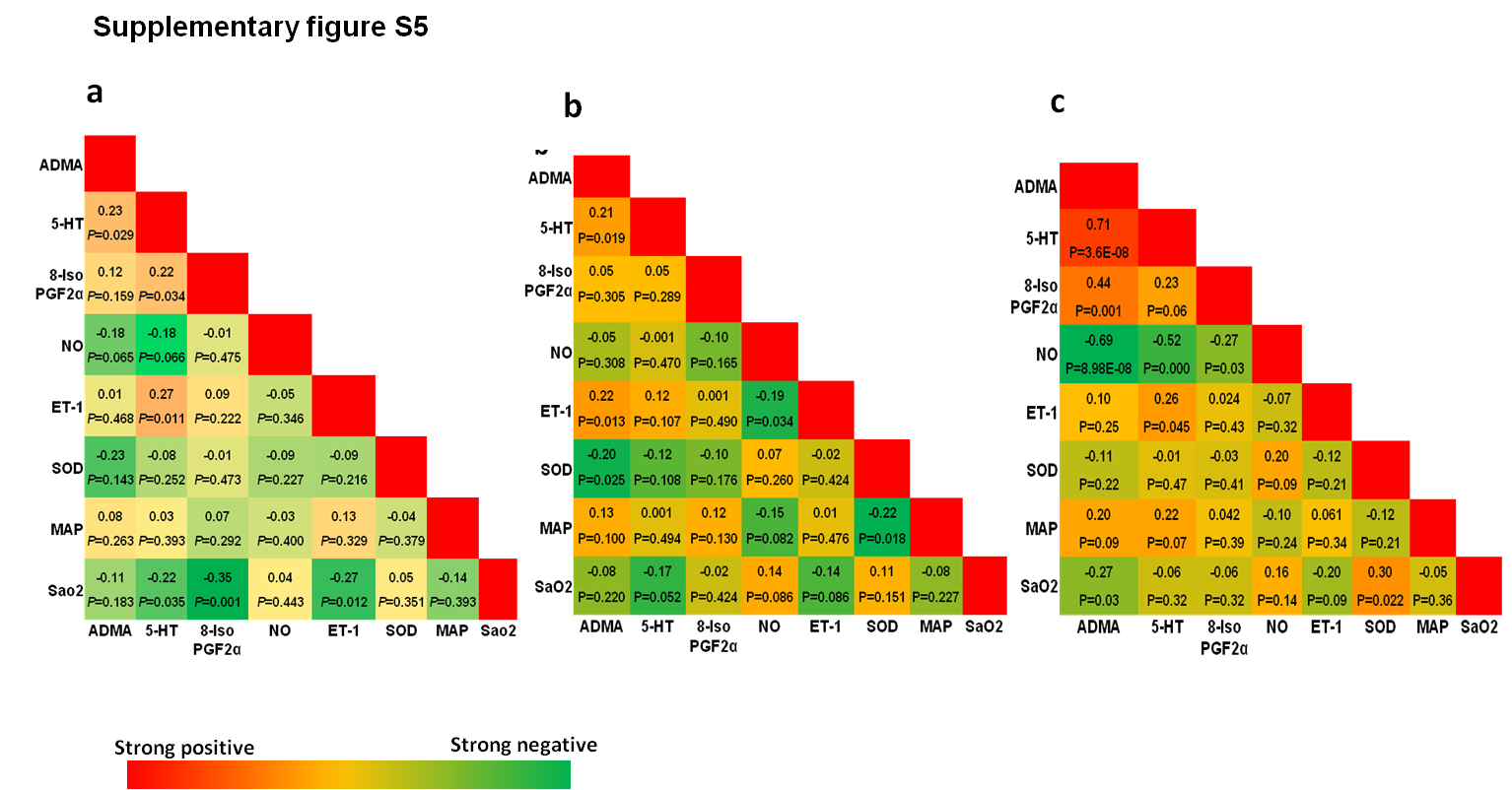

Supplement: Figure S5 — (a–c): Multiple regression analysis in the three groups i.e. HAPE-p, HAPE-f and HLs. The boxes with shades of red depicted positive correlations and boxes with shades of green depicted inverse correlations. Each box contains the coefficient of correlation along with p value. The p value was calculated placing one biomarker against all the studied biomarkers. (TIF) [file pone.0044049.s005.tif]
