# Supplementary material for: Interactions among Vascular-Tone Modulators Contribute to High Altitude Pulmonary Edema and Augmented Vasoreactivity in Highlanders
Source: PLoS One. 2012 Sep 11;7(9):e44049. doi: 10.1371/journal.pone.0044049 (PMC3439466; doi:10.1371/journal.pone.0044049)
Supplement: Table S1 — Real-Time PCR conditions for TPH-1 , ET-1 , REN , CYP11B2 , SOD and NOS3 . (DOC) [file pone.0044049.s006.doc]

| **Supplementary Table S1. Real-Time PCR conditions for *TPH-1*, *ET-1*, *REN*, *CYP11B2, SOD* and *NOS3*** | | | |
| --- | --- | --- | --- |
| **Target** | **Primer sequence** | **Cycling conditions** | **Product size (bp)** |
| *Tph-1* | F 5’- TGAACTATAAACATGGAGACCC-3’  R 5’- TGATAAGTAACCAGCCACAG-3’ | I94°C 10', D94°C 15'', A60°C 60'', 40 cy | 253 |
| *ET-1* | F 5’- CCAGAAACAGCAGTCTTAGG -3’  R 5’- TGTTGACCCAAATGATGTCC -3’ | I94°C 10', D94°C 15'', A60°C 60'', 40 cy | 171 |
| *REN* | 5' - CCACTATATCAACCTCATCAAGAC- 3'  5' - TTCACGACATAATCAAACAGCC 3' | I94°C 10', D94°C 15'', A60°C 60'', 40 cy | 203 |
| *CYP11B2* | F 5’- GGCAGAGGCAGAGATGCTG-3’  R 5’- CTTGAGTTAGTGTCTCCACCAGGA-3’ | I94°C 10', D94°C 15'', A60°C 60'', 40 cy | 130 |
| *SOD* | F 5’- ACACCTTCCACTCTGAGGTCTCACC -3’  R 5’- GAAGATCGTCAGGTCAAAGGCGGG -3’ | I94°C 10', D94°C 15'', A60°C 60'', 40 cy | 280 |
| *NOS3* | F 5’- TGTATG GATGAGTATGACGTGG-3’  R 5’- CGGATC TTATAACTCTTGTGC TG-3’ | I94°C 10', D94°C 15'', A60°C 60'', 40 cy | 181 |
| F = forward; R = reverse; I = initial denaturation; D = denaturation; A = annealing; cy = cycles. | | | |
